# Unexpected Implication of SRP and AGO2 in Parkinson’s Disease: Involvement in Alpha-Synuclein Biogenesis

**DOI:** 10.3390/cells10102792

**Published:** 2021-10-18

**Authors:** Sarah M. Hernandez, Elena B. Tikhonova, Kristen R. Baca, Fanpeng Zhao, Xiongwei Zhu, Andrey L. Karamyshev

**Affiliations:** 1 Department of Cell Biology and Biochemistry, Texas Tech University Health Sciences Center, Lubbock, TX 79430, USA; Sarah.Kader@ttuhsc.edu (S.M.H.); elena.tikhonova@ttuhsc.edu (E.B.T.); bacakristenr@gmail.com (K.R.B.); 2 Center for the Integration of STEM Education and Research (CISER), Texas Tech University, Lubbock, TX 79409, USA; 3 Department of Pathology, Case Western Reserve University, Cleveland, OH 44106, USA; fxz111@case.edu (F.Z.); Xiongwei.Zhu@case.edu (X.Z.)

**Keywords:** alpha-synuclein, signal recognition particle (SRP), Argonaute 2 (AGO2), Parkinson’s disease, Synucleinopathies, protein biogenesis, substantia nigra, neurodegenerative diseases

## Abstract

Parkinson’s disease (PD) is a neurodegenerative disorder classified by the loss of dopaminergic neurons in the substantia nigra pars compacta, the region of the brain that is responsible for motor control. Surviving neurons in this region contain aggregated protein alpha-Synuclein (αSyn) in the form of cytoplasmic inclusions, referred to as Lewy bodies. Changes in αSyn expression are also associated with PD and its progression. Previously, we demonstrated that signal recognition particle (SRP) and Argonaute 2 (AGO2) proteins are involved in protein quality control at the ribosome during translation. We also demonstrated that SRP has an mRNA protection function in addition to a protein targeting function, thus controlling mRNA and protein expression. In this study, we tested involvement of these factors in αSyn biogenesis. We hypothesize that loss of these factors may interfere with αSyn expression, and subsequently, be associated with PD. Using depletion assays in human cell culture and analysis of these proteins in the brains of deceased PD patients, we demonstrate that SRP and AGO2 are involved in the control of αSyn expression and AGO2 has reduced expression in PD. We show for the first time that SRP is involved in mRNA protection of αSyn, a protein that does not have a signal sequence or transmembrane span. Our findings suggest that SRP may interact with a hydrophobic domain in the middle of αSyn during translation. Understanding the molecular mechanisms controlling αSyn biogenesis in cells is vital to developing preventative therapies against PD.

## 1. Introduction

Synucleinopathies are a group of neurodegenerative disorders that are characterized by the accumulation of aggregated forms of alpha-Synuclein (αSyn) in the cytosol of neurons and glial cells [1]. Parkinson’s disease (PD), dementia with Lewy bodies, and Multiple System Atrophy are all forms of Synucleinopathies. PD is the second most prevalent neurodegenerative disorder, after Alzheimer’s disease, and the 18th leading cause of the death in the world as of 2017 [2,3]. PD is characterized by the death of dopaminergic neurons in the substantia nigra pars compacta (SNc) and accumulation of αSyn, seen as Lewy bodies, in surviving neurons [4]. The SNc is the region of the brain responsible for motor control. Therefore, loss of neurons within the SNc (as in the case of PD) results in a loss of motor control. Current therapies for PD rely on the supplementation of dopamine back into the brain. However, these approaches only slow the disease progression, and do not prevent or cure disease.

Current approaches to study pathological αSyn in PD include in vitro αSyn aggregation assays and in vivo mouse models that mimic PD through genetic manipulation or the addition of neurotoxins that induce selective degeneration of dopaminergic neurons [5,6]. These studies focus primarily on factors that affect aggregation kinetics, spread, and clinical outcomes, such as the presence of clinical mutations and the concentration of αSyn. Our approach concentrates on the vital interactions that occur with a protein during its biogenesis. Protein biogenesis explores the factors involved in the correct synthesis, folding, trafficking, modification, secretion, and degradation of a protein. αSyn is localized in the cytosol, cellular organelles (nucleus, endoplasmic reticulum (ER), Golgi, and mitochondria), and extracellular vesicles [7]. Due to its wide localization in and out of the cell, αSyn may interact with a number of factors at different times during its lifespan depending on environmental cues. Previously studied players in αSyn protein biogenesis are molecular chaperones HSPA8, DNAJB1, DNAJB6, HSPA4, CRYAB, DJ-1, and proSAAS and modifying enzymes responsible for αSyn phosphorylation, acetylation, SUMOylation, and ubiquitination [7,8,9]. 

Recently, we discovered a novel protein quality control pathway termed the Regulation of Aberrant Protein Production (RAPP) [10,11]. The pathway monitors protein interactions at the ribosome during translation and degrades their mRNAs if these interactions are disrupted. RAPP prevents accumulation of potentially hazardous mislocalized or misfolded proteins in the cytosol through specific mRNA degradation. Pathological RAPP activation is associated with multiple human diseases [12,13,14,15]. 

Two components of the RAPP machinery are currently determined, Signal Recognition Particle (SRP) and Argonaute 2 (AGO2). SRP conducts mRNA protective function during RAPP in addition to its earlier well-established role as a major protein targeting factor in the cell. SRP, as a targeting factor, is responsible for the first steps in protein transport and correct localization to the ER, plasma membrane, or outside of the cell [16,17]. Previously, we have shown that depletion of the SRP54 subunit of SRP results in a loss of its mRNA protective function, activates the RAPP pathway, and leads to a reduction in the expression of secretory proteins and their mRNAs. This process is facilitated by the engagement of AGO2 protein in close proximity to a nascent polypeptide at the ribosome polypeptide exit site [10]. Overall, our findings demonstrated that SRP and AGO2 are involved in the regulation of mRNA and protein expression of secretory proteins and suggested that they may control expression of other proteins as well. 

In this study, we hypothesize that the components of the RAPP pathway, SRP and AGO2, are involved in the regulation of αSyn biogenesis. To test this hypothesis, we examined αSyn expression in cultured human cells where SRP and AGO2 were depleted. To verify that our findings are relevant to PD, we have tested SRP and AGO2 levels in postmortem PD brains. Overall, this study demonstrates the involvement of SRP and AGO2 in αSyn biogenesis and suggests dysregulation of RAPP during PD. 

## 2. Materials and Methods

### 2.1. Cell Culture, αSyn Expressing Plasmid and Transfections

HeLa Tet-On cells (Clontech (Takara Bio USA), San Jose, CA, USA) were cultured in Dulbecco’s Modified Eagle Medium (DMEM, Sigma, St. Louis, MO, USA, catalog number D6429) with 10% fetal bovine serum and 1% Penicillin/Streptomycin at 5% CO_2_, 37 °C. Cells were plated at 0.5–0.7 × 10^5^ cells/mL 20–24 h prior to transfection of siRNA with Lipofectamine RNAiMAX (Invitrogen (Thermo Fischer Scientific), Waltham, MA, USA, catalog number 13778150). siRNAs specific for AGO2 [18], SRP54 and αSyn (gene *SNCA*) are presented in Supplementary Table S1. Negative control siRNA used as control in AGO2 siRNA transfections was from Thermo Fisher Scientific (catalog number AM4611). Plasmid pCS2-SNCA (see below for details of αSyn cloning) expressing αSyn (0.25 ng/μL in 6-well, 0.2 ng/μL in 12-well) was transfected with Lipofectamine 3000 (Invitrogen, catalog number L3000015) into cells 20–24 h after siRNA transfection. Cells were analyzed 48–72 h after siRNA transfection. For αSyn plasmid titration experiments (shown in Supplementary Figure S1C–E) cells were plated at 2 × 10^5^ cells/mL 20–24 h prior to transfection with the plasmid pCS2-SNCA (0–1.5 ng/μL in 6-well or 12-well plates) and analyzed 48 h after transfection. Template plasmid for αSyn cloning was a gift from Philip J. Thomas (UT Southwestern Medical Center). To clone αSyn into modified pCS2 vector, PCR product containing wild type recombinant αSyn with flanking restriction sites FseI and AscI was generated using Phusion High-Fidelity DNA Polymerase (Thermo Fisher Scientific, F-530S) following by ligation with the vector fragment using T4 DNA ligase (NEB, Ipswich, MA, catalog number M0202S) and amplification in *E.coli* DH5α. Purification of PCR product and plasmid DNA were performed by NucleoSpin Gel and PCR Clean-up kit (Takara Bio USA, San Jose, CA, USA, catalog number 740609) and NucleoSpin Plasmid Transfection-grade kit (Takara Bio USA, catalog number 740490) correspondingly. The construct was verified by DNA sequencing and named pCS2-SNCA. 

### 2.2. Actinomycin D Treatment Experiments

HeLa Tet-On cells were plated and transfected with siSRP54 and pCS2-SNCA plasmid as described above. Cells were treated with DMSO (control) or 8 µg/mL of Actinomycin D 12 h after plasmid transfection for 0, 4, 6, 8, and 10 h. Total RNA was extracted and purified using the NucleoSpin RNA purification kit (Takara Bio USA, 740955) at the indicated time points after treatment. OmpA mRNA (20 ng) was added prior to purification and used for normalization [19]. Relative mRNA levels were quantified using RT-qPCR, as described below.

### 2.3. Western Blotting

Total cell proteins were extracted using Lysis buffer (50 mM Tris pH 7.4, 150 mM NaCl, 1% Triton X-100, 10% glycerol, EDTA-free protease inhibitors (Roche, Basel, Switzerland, catalog number 04693159001) followed by sonication. For αSyn visualization, proteins were separated on a 15% SDS-PAGE and transferred to 0.2 μm PVDF membrane. Membrane was fixed for 30 min with 0.4% Paraformaldehyde in PBS [20]. Blocking was performed in 5% milk in Tris-Buffered Saline with 0.05% Tween 20 (TBS-T-0.05), all antibody incubations and washes were carried out in 1% milk with TBS-T-0.05. αSyn was detected with Syn202 primary antibody (dilution 1:5000; Invitrogen, catalog number 32-8200) and secondary goat anti-mouse HRP. To analyze SRP54, AGO2 and beta-Actin expression, total cell proteins were separated on a 12% SDS-PAGE and transferred to 0.45 μm PVDF membrane. Blocking and antibody incubations were performed in 5% milk in Tris-Buffered Saline with 0.1% Tween 20 (TBS-T). All washes were performed in TBS-T. Primary antibodies used were mouse anti-SRP54 (dilution 1:5000; BD Biosciences, East Rutherford, NJ, USA, catalog number 610940), mouse beta-Actin (dilution1:30,000; ProteinTech, Rosemont, IL, USA, catalog number 66009-1-Ig), rabbit anti-AGO2 (dilution 1:1000; Cell Signaling, Danvers, MA, USA, catalog number 2897S). Secondary antibodies used were goat anti-mouse HRP (dilution 1:30,000; Jackson Laboratories, Bar Harbor, ME, USA, catalog number 115-035-003) and goat anti-rabbit HRP (dilution 1:5000; Jackson Laboratories, catalog number 111-035-003). 

### 2.4. Microscopy

HeLa Tet-On cells were plated at 0.5–0.7 × 10^5^ cells/mL in 6 well plates with 13 mm glass coverslips. Cells were transfected with siRNAs and then with a plasmid as marked in the figures. Cells were fixed in 4% paraformaldehyde in PBS for 15 min and incubated in permeabilization buffer (0.2% Triton X-100, 3% BSA in PBS) for 20 min at 4 °C. Primary antibodies were prepared in permeabilization buffer and were added to coverslips for 1 h at room temperature. Primary antibodies used were Syn202 (dilution 1:500; Invitrogen, catalog number 32-8200) and mouse anti-SRP54 (dilution 1:1000; BD Biosciences, catalog number 610940). Secondary antibody prepared in permeabilization buffer was added to coverslips for 30 min in the dark at room temperature. Alexa Fluor 555 goat anti-mouse IgG (dilution 1:500; LifeTechnologies, Carlsbad, CA, USA, catalog number A21422) was used as a secondary antibody. All washes were performed in washing buffer (0.2% Triton X-100 in PBS). Nuclei were stained with DAPI (Invitrogen, catalog number D1306) for 3–4 min at room temperature. Coverslips were mounted onto microscopy slides with Prolong Gold antifade reagent (Invitrogen, catalog number P36930). Microscopy images were obtained using Nikon Ti-E confocal microscope (Nikon, Tokyo, Japan). Corrected total cell fluorescence (CTCF) is expressed in relative fluorescence units and calculated as CTCF = Integrated Density − (Area of selected cell × Mean fluorescence of background readings). All measurements for CTCF calculations were performed using Image J 1.53a. 

### 2.5. RNA Purification, cDNAs, Real-Time Quantitative PCR (RT-qPCR)

Total RNA was extracted from HeLa Tet-On cells using Trizol (Invitrogen, catalog number 15596026) and purified according to company protocol. cDNA was generated using High Capacity cDNA Reverse Transcription Kit (Applied Biosystems (Thermo Fisher Scientific), 4368814). Sequences of primers used for RT-qPCR are shown in Supplementary Table S2. RT-qPCR was performed using Quant Studio 12 K Flex Real-Time PCR System with Power SYBR Green PCR Master Mix (Applied Biosystems, 4367659). Comparative C_T_ method was used to quantify the qPCR results [21]. 

### 2.6. Human Substantia Nigra Collection and Analysis

Human midbrain tissue samples were collected at time of autopsy and stored at −80 °C. Samples were obtained from the University Hospital Case Medical Center under an approved IRB protocol or from the Harvard Brain Tissue Resource Center through the Neurobiobank program. For this study, a small area of the substantia nigra from PD patients (*n* = 7) and age-matched control patients (*n* = 6) was carefully dissected out and homogenized with RIPA lysis buffer plus protease inhibitor mixture. Characteristics of the PD and control samples are presented in Supplementary Table S3. The study was conducted in accordance with the Declaration of Helsinki, and the protocol was approved by The University Hospitals Institutional Review Board (03-00-26) on 4 January 2020.

### 2.7. Statistical Analysis

All statistical analyses were performed using Prism 8 by GraphPad. Statistical methods, number of repeats (*n*), and *p* values are indicated within the figure legends. 

## 3. Results

### 3.1. αSyn Expression in Cultured Human Cells

To study the regulation of αSyn biogenesis in human cells, we developed a cell culture model to express αSyn and optimize conditions for cell growth, siRNA, and plasmid transfections using HeLa Tet-On cell line. We demonstrate that protein expression of endogenous αSyn in this cell line is not detectable (Supplementary Figure S1A). No specific product was observed at the 15 kDa range corresponding to monomeric form of αSyn and a high molecular weight band is a nonspecific product, see below. This feature makes HeLa Tet-On an appropriate model to exogenously overexpress αSyn with minimal background levels. When αSyn is expressed from a plasmid in this cell line and analyzed by Western blotting using the Syn202 antibody, there are two major bands observed: one band around the expected molecular weight (15 kDa) and a high molecular weight (HMW) species at the top of the gel (>250 kDa, Supplementary Figure S1A). To verify that the protein detected by the Western blot is indeed αSyn, we used αSyn specific siRNA knockdown. Transfection with αSyn siRNA leads to a complete loss of the 15 kDa band, while the >250 kDa band remained. The loss of αSyn expression is also seen on the mRNA level by RT-qPCR, confirming the specificity of mRNA expression to the αSyn plasmid and effectiveness of the siRNA (Supplementary Figure S1B). To further verify that the HMW species are not specific to αSyn, HeLa Tet-On cells were transfected with an increasing amount of αSyn expressing plasmid, pCS2-SNCA, and the αSyn expression was analyzed both on the protein and mRNA level 48 h after transfection. Only the 15 kDa band increases with increasing plasmid concentration, while the HMW species remain the same, confirming one more time that the HMW species are not relevant to a recombinant αSyn (Supplementary Figure S1C). These results were consistent over several biological repeats, with αSyn protein at 15 kDa following a linear increase in expression with increasing concentration of the plasmid transfected, up to 0.75 ng/μL (Supplementary Figure S1D). A similar pattern was seen through analyzing mRNA using RT-qPCR (Supplementary Figure S1E). For subsequent experiments in this paper, only the specific 15 kDa band detected on Western blots was analyzed for αSyn protein expression.

### 3.2. Depletion of SRP54 Affects αSyn Expression

**Figure 1:**
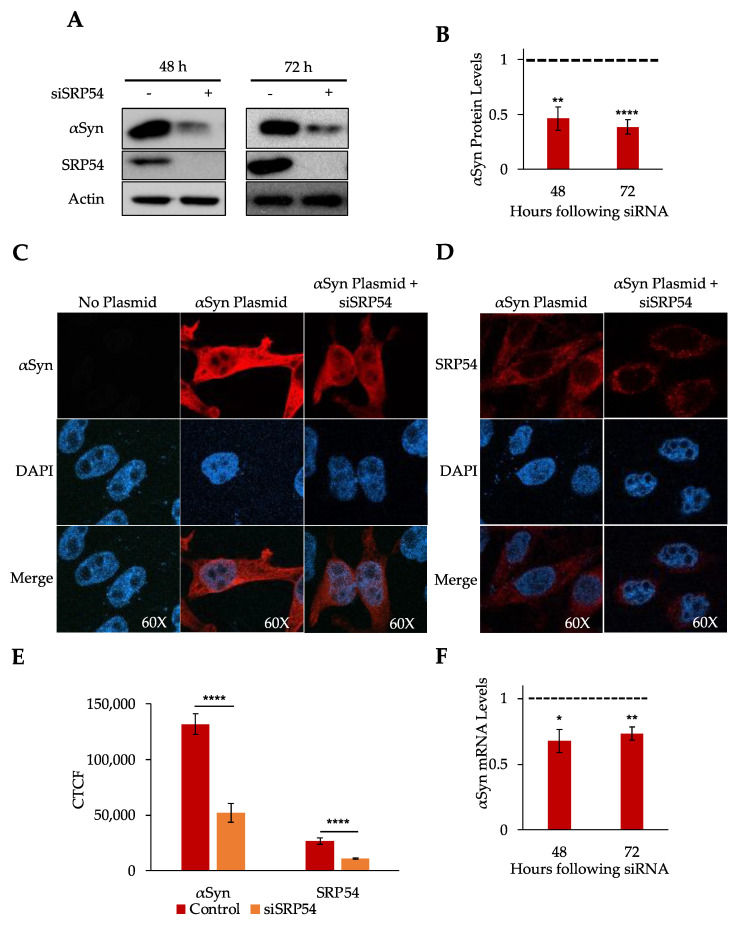
SRP Regulates αSyn expression in cultured human cells. SRP54 knockdown leads to decrease in αSyn protein and αSyn mRNA expression in cultured human cells as detected by Western blot (**A**,**B**), immunofluorescence (**C**–**E**), and RT-qPCR (**F**). (**A**) Western blot analysis of total cell lysates using antibodies against αSyn, SRP54, and beta-Actin are shown. siSRP54 (siRNA specific for SRP54) was transfected into HeLa Tet-On cells, 24 h later, αSyn plasmid was transfected. Cells were analyzed 48 or 72 h post siRNA transfection. (**B**) Quantification of αSyn Western blots using Image J. αSyn levels were normalized to beta-Actin protein levels and then presented in a graph relative to αSyn protein levels in control cells taken as 1 in the respective time point. Black dashed line indicates αSyn protein levels in control cells. Graph shows mean values ± SE with *n* = 6 independent experiments at 48 h and *n* = 13 independent experiments at 72 h after siRNA transfection. (**C**) Immunofluorescence reveals decrease of αSyn expression in cultured human cells upon SRP54 depletion. Cells were transfected with siSRP54, and after 24 h with αSyn expressing plasmid (or mock transfected in controls). Confocal microscopy of αSyn in HeLa Tet-On cells was conducted 48 h after SRP54 siRNA was transfected. αSyn (shown in red) was detected with αSyn antibody and with Alexa 555 secondary antibody, nucleus stained with DAPI (shown in blue). Images are shown at 60× magnification. (**D**) Depletion of SRP54 expression following siRNA knockdown in HeLa Tet-On cells as observed by confocal microscopy. SRP54 (shown in red) was detected in the cells expressing αSyn in siSRP54 treated or control cells 48 h after siSRP54 was transfected. SRP54 antibody was used with Alexa 555 secondary antibody, nucleus stained with DAPI (shown in blue). Images shown at 60× magnification. (**E**) Corrected total cell fluorescence (CTCF) is expressed in relative fluorescence units and calculated as CTCF = Integrated Density − (Area of selected cell × Mean fluorescence of background readings). All measurements for CTCF calculations were performed in Image J. Graph shows mean values ± SE. *n* = 39 cells for αSyn mock samples, *n* = 19 cells for αSyn with siSRP54 samples, *n* = 18 cells for SRP54 mock and siSRP54 samples. (**F**) αSyn mRNA is downregulated in SRP54 knockdown cultured human cells. Quantification of mRNA expression levels at 48 and 72 h after SRP54 siRNA transfection is shown. mRNA levels measured by RT-qPCR, normalized to beta-Actin mRNA levels and presented relative to αSyn mRNA levels in control cells (black dashed line indicates αSyn mRNA levels in control cells). Graph shows mean values ± SE with a total of 9 independent experiments at 48 h and 12 independent experiments at 72 h after siRNA transfection. Significance determined by paired *t* test for protein and mRNA, * *p* < 0.05, ** *p* < 0.01, **** *p* < 0.0001. Significance determined by unpaired *t* test with Welch’s correction for corrected total cell fluorescence, **** *p* < 0.0001.

During protein synthesis, the nascent chain that emerges from the ribosomal tunnel interacts with a number of proteins involved in the correct folding, translocation, and modification of the synthesizing protein. In eukaryotes, secretory proteins are targeted to the ER by the Signal Recognition Particle (SRP) [16,17]. SRP recognizes the signal sequences, additional regions on the N-termini of the emerging secretory protein polypeptide nascent chains, during translation. The ability of SRP to recognize this region is based on its hydrophobicity [10,22]. Although αSyn does not have a signal sequence, it does contain a hydrophobic region known as the non-amyloid beta component (NAC) region present in the middle of the protein. Our recent study demonstrated that the hydrophobic core of a signal sequence is major recognition element for SRP [22]. Recent research in yeast showed that SRP is essential for the translocation of proteins that contain hydrophobic transmembrane domain regions to the plasma membrane [23]. The SRP dependence of these proteins was not affected by the relative location of the transmembrane domains to the N-terminus. SRP also has an mRNA protective function during the RAPP protein quality control in addition to its protein targeting function. Therefore, we intend to verify if SRP is capable of recognizing αSyn, a protein with no signal sequence, but a hydrophobic region, and if it is involved in αSyn mRNA protection from degradation. For this study, we focus on SRP54, the SRP subunit that binds to the signal sequence of the synthesizing protein and was previously shown to be necessary for the biogenesis of secretory proteins and for protection of their mRNA from degradation [10,14]. To determine if SRP54 is involved in αSyn biogenesis, SRP54 was knocked down to hinder subsequent SRP function. siSRP54 (siRNA specific to SRP54) was transfected into HeLa Tet-On cells, followed by transfection of αSyn plasmid 24 h later. Total cell protein and RNA were extracted 48 and 72 h after siRNA transfection for the analysis by Western blot and RT-qPCR, respectively. We achieved very efficient SRP54 depletion–no SRP54 was detected by Western blot even after 48 h, beta–Actin was used as a loading control (Figure 1A). Remarkably, SRP54 depletion leads to a significant decrease in αSyn protein expression (50–60%), as shown by Western blotting with the Syn202 antibody (Figure 1A,B). To further verify the effect of the SRP54 knockdown on αSyn expression, αSyn levels were analyzed by immunofluorescence 48 h after siSRP54 transfection. HeLa Tet-On cells with or without siSRP54 and αSyn plasmid were stained for evaluation of the αSyn protein (Figure 1C) and for SRP54 to verify its knockdown (Figure 1D). As expected, there is a decrease in SRP54 protein level with siSRP54 transfection as compared to the cells without siRNA (Figure 1D). As visualized with confocal microscopy, there is no detectable αSyn signal in cells without αSyn plasmid (Figure 1C). There is a significant decrease in αSyn protein level with siSRP54 as compared to cells expressing αSyn without siRNA. The decrease in αSyn protein observed by immunofluorescence is significant as demonstrated by the quantification of the corrected total cell fluorescence (CTCF) of multiple cells on the same coverslip using Image J (Figure 1E). These data are consistent with those of the Western blot analysis (Figure 1A,B). Previously, it was shown that substrates of SRP have reduced levels of both protein and mRNA levels when SRP54 is depleted, implicating SRP in mRNA protection [10,14]. To determine if the loss of SRP54 affects mRNA levels of αSyn, we have used RT–qPCR to analyze 48 and 72 h after siSRP54 transfection. αSyn mRNA was downregulated by 30–40% after already 48 h after SRP54 was depleted (Figure 1F). Overall, our data demonstrate a significant effect of SRP54 depletion on the mRNA and protein expression of αSyn.

**Figure 2:**
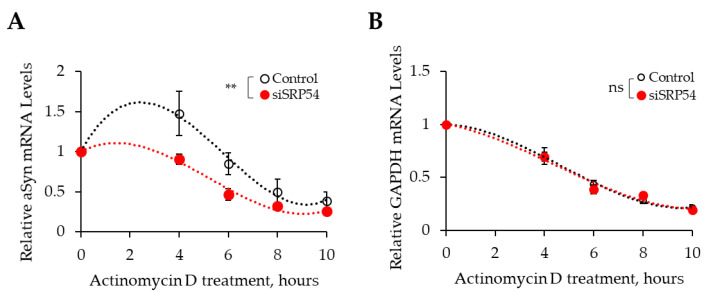
Decrease in αSyn mRNA following SRP54 knockdown is due to specific mRNA degradation. mRNA was quantified for both αSyn (**A**) and GAPDH (**B**) at 0, 4, 6, 8, and 10 h after beginning of the Actinomycin D treatment in siSRP54 transfected and in control cells. mRNA levels were measured by RT-qPCR, normalized as described in Material and Methods and presented relative to respective mRNA levels at timepoint 0 (set at 1 for both, the control and the siSRP54 treated cells). Graphs show mean values ± SE with a total of 3 independent experiments; analysis: two-way ANOVA, ** *p* < 0.01, ns—not significant. Trendlines were used to present the dynamics of mRNA level changes during Actinomycin D treatment.

Decrease in mRNA levels can be caused by reducing mRNA synthesis or by increasing mRNA degradation. As we demonstrated earlier SRP has a protective function on its substrates’ mRNAs of secretory proteins to prevent their degradation. Loss of SRP54 interaction due to mutations in the signal sequences of secretory proteins leads to a decrease in their mRNA levels through mRNA degradation [14]. On the basis of these observations, we hypothesized that αSyn mRNA depletion is caused by its mRNA degradation. To test this hypothesis, we performed an Actinomycin D treatment experiment. Actinomycin D is an antibiotic that inhibits RNA synthesis by stabilizing double-stranded DNA structure, preventing RNA polymerase to bind [24]. Cells were treated with Actinomycin D 12 h after αSyn plasmid transfection. Total cell RNA was extracted after 0, 4, 6, 8, and 10 h of Actinomycin D treatment and mRNA levels were determined by RT-qPCR (Figure 2). Indeed, there is a significant decrease of the αSyn mRNA level in siSRP54 treated cells in comparison with control cells in the presence of Actinomycin D (Figure 2A). This effect is specific to αSyn, as there is no change in the dynamic of degradation of mRNA of glyceraldehyde-3-phosphate dehydrogenase (GAPDH), the protein that is not targeted by SRP (Figure 2B). Based on these results, we conclude that αSyn is a substrate of SRP and that loss of SRP54 results in a loss of the protective function and subsequent αSyn mRNA degradation.

### 3.3. Argonaute 2 Regulates αSyn Expression

**Figure 3:**
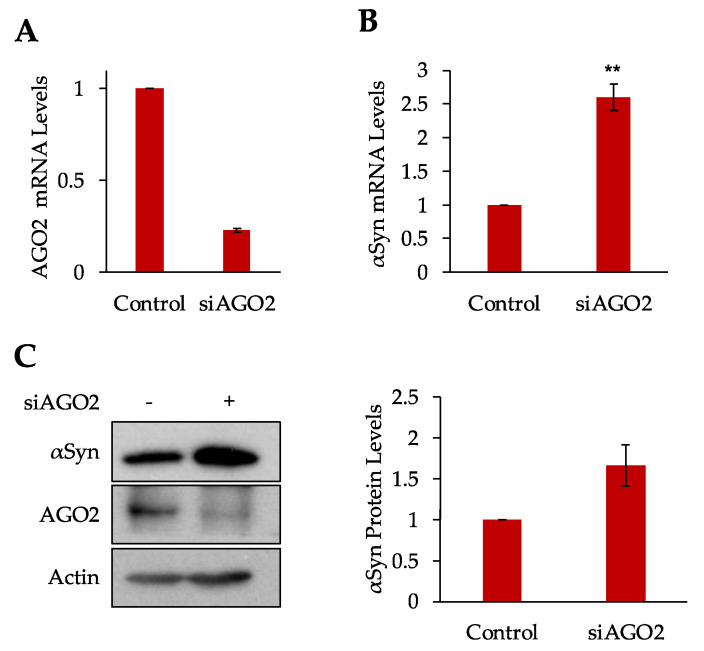
Depletion of AGO2 Leads to an Increase in αSyn Expression. (**A**) AGO2 expression is significantly decreased in the HeLa Tet-On cells treated with siAGO2. AGO2 mRNA levels were measured by RT-qPCR 48 h after siAGO2 transfection, normalized to HPRT mRNA levels and presented relative to AGO2 mRNA levels in control cells. (**B**) Quantification of αSyn mRNA expression levels at 48 h after AGO2 siRNA transfection. mRNA levels measured by RT-qPCR. αSyn mRNA levels were first normalized to HPRT mRNA levels and then to αSyn mRNA levels in control cells. Graph shows mean values ± SE with a total of *n* = 3 independent experiments. (**C**) Western blot of total cell lysate using αSyn, AGO2, and beta-Actin antibodies (left panel). Quantification of αSyn Western blots using ImageJ (right panel). Normalized to beta-Actin protein levels and then to αSyn protein levels in control cells. Graph shows mean values ± SE with a total of *n* = 3 independent experiments. Significance determined by paired *t* test for protein and mRNA, ** *p* < 0.01.

Our findings that SRP is important for αSyn biogenesis, and its mRNA stability suggested that the RAPP pathway controls αSyn during its synthesis on the ribosome. Earlier, on the example of preprolactin, we demonstrated that Argonaute 2 (AGO2) is involved in the RAPP response together with SRP, but it actioned in the opposite direction. While SRP protects preprolactin mRNA from degradation, AGO2 triggers the mRNA decay [10]. When the SRP54 subunit failed to bind to the signal sequence, AGO2 was found in close proximity to the preprolactin polypeptide nascent chain, initiating the RAPP response. This result, directly or indirectly, leads to a decrease in mRNA and protein expression of the synthesizing protein. The mechanism of RAPP and the role of AGO2 in the pathway is not completely understood. AGO2 is most studied for its role in post-transcriptional gene silencing with cleavage of mRNAs and translational repression. However, more recent studies have implicated AGO2 in the regulation of transcription, modification of mRNA, and regulation of translation [25]. We decided to test if AGO2 plays a role in αSyn biogenesis. Considering AGO2 as an antagonist to SRP54 in the RAPP pathway, we hypothesize that AGO2 will affect αSyn in a matter opposite to SRP54. We would expect there would be an increase in αSyn expression when AGO2 is knocked down, due to loss of regulatory control by AGO2. To deplete AGO2, the AGO2 specific siRNAs (siAGO2) were transfected into HeLa cells, followed by αSyn plasmid transfection 24 h later. Total cell protein and mRNA were extracted 48 h after siRNA transfection. AGO2 was efficiently depleted in the cells transfected with siAGO2 as confirmed by RT-qPCR and Western blotting (Figure 3A,C). When AGO2 is knocked down, we observed an increase in αSyn mRNA and protein expression, as shown by RT-qPCR and Western blotting with the Syn202 antibody (Figure 3B,C). This increase was specific to αSyn and did not affect expression of beta-Actin (Figure 3C-left panel). These results demonstrate that AGO2 is involved in αSyn biogenesis, similar to its role in regulating expression of preprolactin. AGO2 works to regulate mRNA and protein expression of target genes in opposite to that seen with SRP54. Although there is a clear role in expression regulation, the mechanism and its biological role is not clear yet at this time. AGO2 has numerous roles within the cell, such as involvement in RNA interference, chromatin remodeling, and transcriptional repression, although it is not known which role it is performing in the RAPP pathway [11,15,25].

### 3.4. Expression of AGO2 Is Reduced in the Substantia Nigra of PD Patients

**Figure 4:**
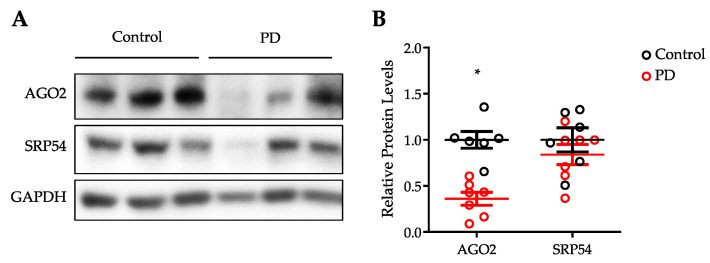
Evaluation of SRP54 and AGO2 protein levels in PD postmortem brains. (**A**) Representative Western blots with whole lysates of substantia nigra from PD and control using AGO2, SRP54, and GAPDH antibodies. (**B**) Quantification analysis of Western blots was performed by Image J and GAPDH was used as an internal loading control. Graph shows mean values ± SE with a total of *n* = 6 for control brains and *n* = 7 for PD brains. Significance determined by independent two-tailed *t* test, * *p* < 0.05.

The results described above are compelling that both SRP54 and AGO2 control αSyn biogenesis in cultured human cells. However, it is important to examine the role of these two proteins in a pathological state to understand if they are implicated in αSyn pathogenesis and disease. Currently, it is understood that αSyn pathology in the brain begins in the olfactory bulb and medulla as seen as Lewy bodies. This pathology spreads through the midbrain in a prion-like manner, eventually encompassing the primary and motor cortices [7]. Although Lewy bodies are seen to spread throughout the brain, the pathological feature of PD is loss of neurons in the SNc. The SNc is a region in the substantia nigra (SN) that is composed of dopaminergic neurons that signal throughout the basal ganglia. The basal ganglia are involved in control of motor function. Loss of these neurons results in loss of motor control, which comprises the most notable clinical symptoms of PD [26]. To determine if the protein levels are altered in the pathological state, the SN of patients with PD were analyzed for the protein levels of SRP54 and AGO2. Samples were analyzed by Western blotting with antibodies against AGO2 and SRP54 (Figure 4A). Western blot of GAPDH was performed as a loading control. Quantification of AGO2 and SRP54 proteins were performed and protein levels were normalized to GAPDH (Figure 4B). As shown in Figure 4, the levels of AGO2 were significantly decreased in PD patients. The levels of SRP54 were also lower in some PD patients but with higher variation. Although this high variation does not allow to make a general strong conclusion about SRP54 levels in the PD patients, the data suggest that SRP54 may be affected in some of them. The data presented in Figure 4 led us to conclusion that AGO2 protein was decreased in brains with PD.

## 4. Discussion

PD is a neurodegenerative disorder characterized by the loss of dopaminergic neurons and the presence of Lewy bodies composed of aggregated αSyn in the SNc. Cases of PD are either familial or sporadic. There are three types of hereditary mutations associated with familial PD: dinucleotide repeat variations in the promoter region, locus multiplications, and missense point mutations. The first two types of mutations occur only to αSyn and result in an increase in αSyn mRNA and protein levels in the brain. Due to the clear association between PD pathology and the increased levels of αSyn with these mutations, PD models that focus on αSyn (in vitro and in vivo) use the overexpression of αSyn to mimic disease pathology. There are 27 genes (αSyn included) with missense mutations that are associated with familial PD [27]. These genes are involved in protein homeostasis, mitochondrial function, and vesicular transport. However, in sporadic cases of PD, which represent majority of PD cases, there is no known direct cause of disease. In familial PD with missense mutations and sporadic PD, there is no consensus on the levels of αSyn mRNA and protein levels in the brain. There are a number of studies exploring αSyn expression in the brains of PD patients; however, there are conflicting conclusions on whether αSyn is increasing, decreasing, or staying the same in different brain region [28,29,30,31]. Therefore, effects of the reduction of αSyn expression with decreased SRP54 and its elevation with decreased AGO2 seen in our study are useful for clinical and pathological research of PD. The increased expression of αSyn when AGO2 is depleted in cell culture combined with the decreased expression of AGO2 in PD patients suggests that AGO2 plays a role in regulating the normal expression of αSyn. Dysregulation of αSyn (resulting in an increased expression) through dysfunction of AGO2 can have detrimental effects in the brain, resulting in PD. Due to the wide range of patient pathology, it is unlikely a decrease in SRP54 or AGO2 is a major factor in every single case. However, studying the role of SRP54 and AGO2 with αSyn is crucial to further the understanding of the underlying molecular mechanism behind PD. Determining how disruption in αSyn biogenesis is implicated in PD will reveal the basic mechanism behind αSyn pathogenesis.

SRP54 is known to interact with the signal sequence of secretory proteins once it is emerged from the ribosome [22,32,33,34]. In addition to proteins that contain a signal sequence, SRP54 is also necessary for the proper translocation of transmembrane proteins through an interaction with the hydrophobic transmembrane domains [23]. The dependence of proper transmembrane protein translocation on SRP54 does not require a transmembrane domain to be near the N-terminus, only that is it not near the C-terminus. Thus, considering that 30–40 amino acid residues are inside of the ribosome tunnel during translation, a hydrophobic domain anywhere along the protein between the first residues of the N-terminus and the 30–40 amino acids from the C-terminus could interact with SRP. Analysis of αSyn hydrophobicity (from αSyn sequence shown in Supplementary Figure S2A) by Kyte-Doolittle plot (ProtScale-ExPASy) shows three peaks with positive hydrophobic score: site I, located outside of NAC domain (amino acids 51–52), and two short hydrophobic stretches within NAC domain-site II (amino acids 69–75) and site III (amino acids 88–92) (Supplementary Figure S2B,C). Evaluating the hydrophobic properties of these domains by GRAVY program revealed that all three domains have marginal hydrophobic properties (hydropathy values around 1.8–1.9). SRP has preferences toward substrates with strong hydrophobic domains, for example preprolactin with GRAVY value of 2.4 [35]. However, it also binds signal peptides with less significant hydrophobicity properties. Hydrophobic region of signal peptide, where SRP binds, is usually 8–25 amino acid long. Site I is too short and introducing charge in this region obviously would decrease hydrophobic score. There is a G51D mutation reported that affects pathology of αSyn protein [36]. It is not known if this mutation might affect the helical structure of N-terminal amphipathic region and/or destabilize interaction with αSyn partners [37]. The sites II and III could be the potential sites for SRP binding if the direct interactions could exist. More studies are needed to clarify this possibility. Studies linking SRP and αSyn have been few. Using an *E. coli* model overexpressing human αSyn, bacterial SRP was found to be necessary for αSyn translocation to the periplasm [38]. In addition, it was shown that the C-terminus of αSyn was necessary for αSyn translocation to the periplasm. Furthermore, a proteomics study in embryonic stem cells found a direct interaction between αSyn and SRP9, a subunit of SRP, although no further functional studies have been performed [39]. SRP9 is responsible for the elongation-arrest of the ribosome during protein synthesis, allowing SRP to properly traffic the synthesizing proteins. Although further experiments are needed to be performed to determine if SRP54 directly interacts with the αSyn NAC hydrophobic region, our study demonstrates that SRP54 controls αSyn expression most likely through αSyn mRNA protection similar to SRP role in the RAPP process. Thus, our findings demonstrate that SRP has wider implication in the biogenesis of different proteins, and it is not limited to secretory and membrane proteins only. This work is the first demonstration that SRP is involved in mRNA protection of αSyn, a protein that does not have a distinct signal sequence or transmembrane span.

AGO2 was originally found to be involved in RAPP pathway through in vitro translation with site-specific photo-crosslinking when the signal sequence was mutated which decreased the binding ability of SRP54 [10]. It was demonstrated that when AGO2 is depleted, the mRNA of the RAPP substrate, preprolactin, was increased. In this study, we demonstrate that AGO2 is involved in αSyn biogenesis, in the same manner as preprolactin. These results represent a novel class of substrates for the RAPP pathway. SRP was previously regarded to only target secretory proteins. However, αSyn is not a typical secretory protein, but is seen throughout the cytoplasm, intracellular organelles, and outside of the cell. In addition, αSyn does not have a typical signal sequence located at the N-terminus, as seen in secretory proteins. Therefore, the involvement of SRP54 is a novel regulatory control mechanism for proteins that do not contain an N-terminal signal sequence. Recently, SRP was shown to be involved in the proper translocation of membrane proteins that contain internal hydrophobic regions [23]. Thus, SRP is involved in the proper biogenesis of more than just secretory proteins, although the mechanisms of this involvement are unknown.

In our cell culture models, we observe an alteration in αSyn mRNA and protein expression when SRP54 or AGO2 are depleted. In addition, there is a significant decrease in AGO2 in the post-mortem brains of patients with PD. SRP54 was also decreased in some patients. Based on these results, it is clear that SRP54 and AGO2 are somehow implicated in PD. Both SRP54 and AGO2 have previously been implicated in neurodegenerative diseases; however, no such connections have been made with PD before our findings [13,40]. Individual genetics and symptoms of patients from the post-mortem brain samples are unknown, making us unable to identify if there is any relationship between SRP54 or AGO2 expression and clinical presentation. Future studies are needed to understand exactly how these two proteins are implicated in PD and how they are involved in αSyn expression.

Overall, this study is significant due to its insight into the early biogenesis of αSyn. Taking a closer look into the biogenesis of αSyn, such as co-translational interacting partners, mRNA and protein regulation, and post-translational interacting partners, is key to understanding how and why αSyn is implicated in PD. This study provides a connection between proteins involved in αSyn biogenesis and PD. Understanding the initial mechanisms in the disease process provides a framework for preventative therapies for PD that will be able to treat the disease, not just minimize the symptoms.

## Data Availability

All data are presented in this publication and in the Supplementary Materials associated with it.

## Supplementary Materials

The following are available online at https://www.mdpi.com/article/10.3390/cells10102792/s1, Supplementary Figure S1: Expression of Recombinant αSyn in Tet-On Cells, Supplementary Figure S2: Hydrophobic Domains of αSyn Protein, Supplementary Table S1: Sequences of siRNAs Used for Targeted Knockdown, Supplementary Table S2: Sequences of Primers Used for RT-qPCR Analysis, Supplementary Table S3: Demographic Characteristics of Substantia Nigra Samples.
