# Supplementary material for: Unexpected Implication of SRP and AGO2 in Parkinson’s Disease: Involvement in Alpha-Synuclein Biogenesis
Source: Cells. 2021 Oct 18;10(10):2792. doi: 10.3390/cells10102792 (PMC8534902; doi:10.3390/cells10102792)
Supplement: Supplementary file 1 [file cells-10-02792-s001.zip › cells-1396909-supplementary.pdf]

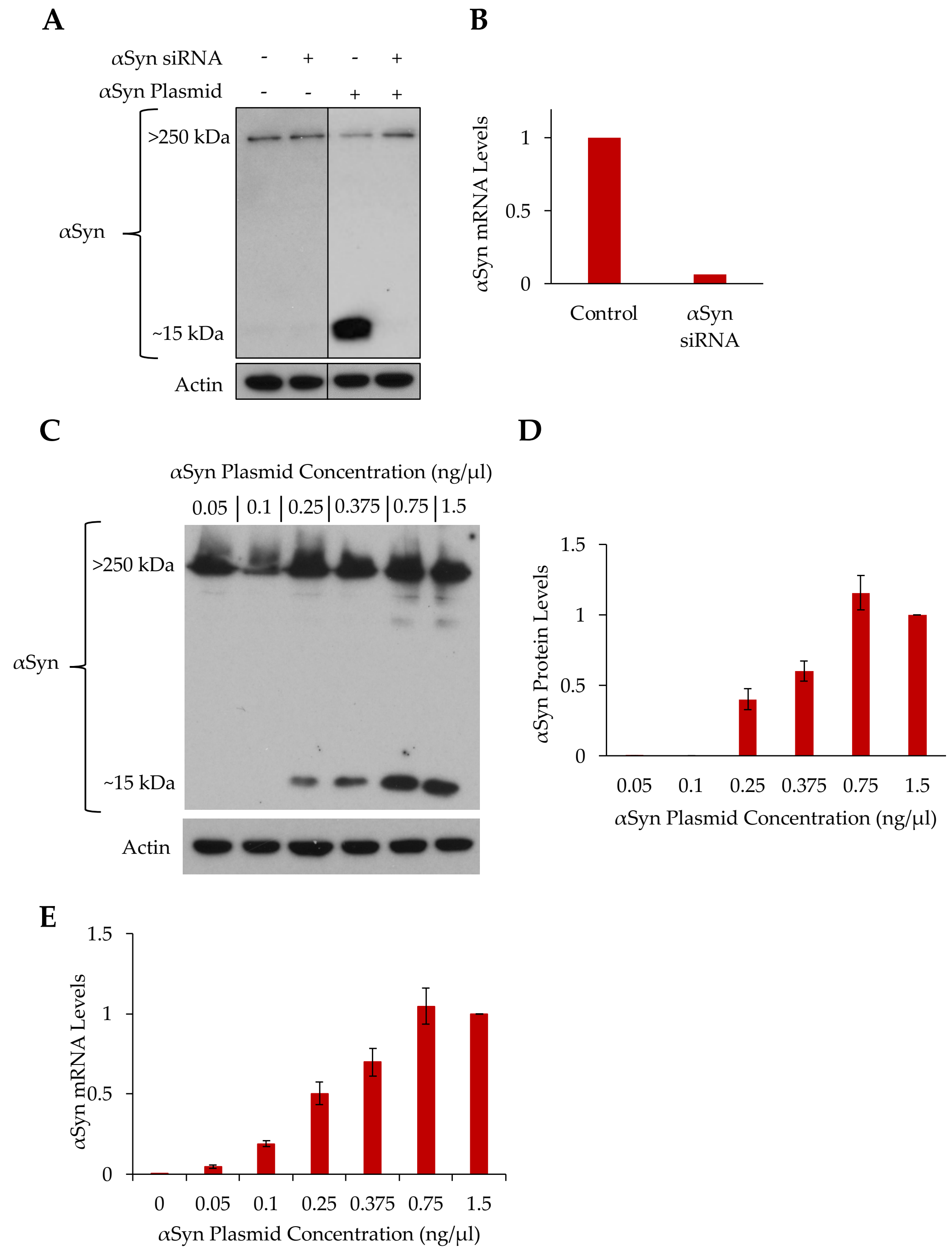

Supplementary Figure S1. Expression of Recombinant αSyn in Tet-On Cells. (**A**) Western blot of αSyn protein (with or without recombinant αSyn) following knockdown with αSyn specific siRNA. 15 kDa band is the monomer, >250 kDA band is nonspecific. αSyn plasmid is pCS2-SNCA. beta-Actin detected by western blot is a loading control. (**B**) RT-qPCR analysis of mRNA levels of recombinant αSyn in the cells treated with αSyn specific siRNA and in the control cells. (**C**) Western blot of αSyn protein following transfection with increasing concentration of the pCS2-SNCA plasmid (0.05-1.5 ng/μl). (**D**) Quantification of western blots using ImageJ. Normalized to beta-Actin protein levels and then to αSyn protein levels at 1.5 ng/μl concentration. Graph shows mean values ± SE with a total of n= 3 independent experiments. (**E**) RT-qPCR analysis of αSyn mRNA levels following transfection with increasing concentration of the pCS2-SNCA plasmid (0-1.5 ng/μl). Normalized to beta-Actin mRNA levels and then to αSyn mRNA levels at 1.5 ng/μl concentration. Graph shows mean values ± SE with a total of n= 3 independent experiments.

A

10	20	30	40	50
**MDVFMKGLSK**	**AKEGVVAAAE**	**KTKQGVAEAA**	**GKTKEGVLYV**	**GSKTKEGVVH**
60	70	80	90	100
**GVATVAEKTK**	**EQVTNVGGAV**	**VTGVTAVAQK**	**TVEGAGSIAA**	**ATGFVKKDQL**
110	120	130	140	
**GKNEEGAPQE**	**GILEDMPVDP**	**DNEAYEMPSE**	**EGYQDYEPEA**	

B

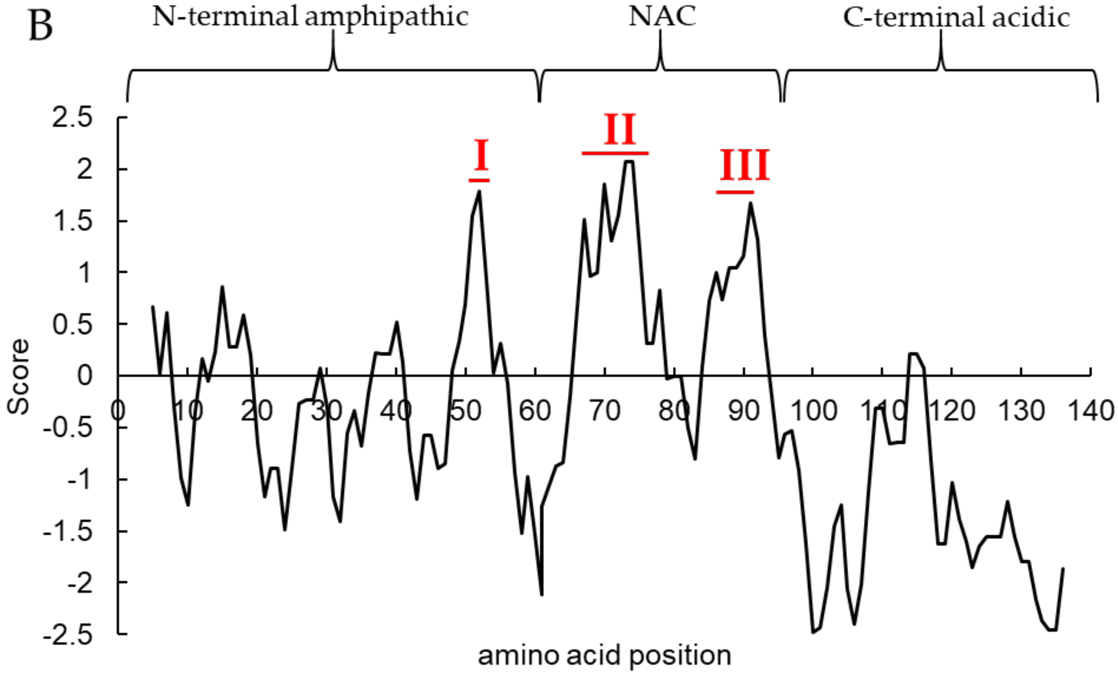

C

Site (as labeled on panel B)	Amino acid Position	Amino acid sequence	GRAVY value
I	51-52	GV	1.9
II	69-75	AVVTGVT	1.8
III	88-92	IAAAT	1.84

Supplementary Figure S2. Hydrophobic Domains of αSyn Protein. (**A**) Amino acid composition of αSyn. (**B**) Kyte-Doolittle hydropathy plot of full length αSyn. Hydrophobicity score was calculated using https://web.expasy.org/protscale/. Domains with positive hydrophobic score are labeled as I, II and III. αSyn structural domains are amphipathic (1-60), non-amyloid beta component, NAC (61-95) and acidic domain (96-140 amino acids). (**C**) GRAVY values of selected hydrophobic domains. Average hydropathy value of possible interacting domains were calculated using http://www.gravy-calculator.de/.

Supplementary Table S1. Sequences of siRNAs Used for Targeted Knockdown.

siRNA	Target NM	Sequence	Company	Concentration Used (nM)
siSNCA (αSyn)	NM_000345.3	5′-UGGCAACAGUGGCUGAGAA-3′	Dharmacon	11.25
siSRP54	NM_003136.4	5′-GAAAUGAACAGGAGUCAAUdTdT-3′	Dharmacon	13.75
siAGO2-1	NM_012154	5′-GCACGGAAGUCCAUCUGAAUU-3′	Dharmacon	10
siAGO2-2	NM_012154	5′-GCAGGACAAAGAUGUAUUAUU-3′	Dharmacon	10
siAGO2-3	NM_012154	5′-GGGUCUGUGGUGAUAAAUAUU-3″	Dharmacon	10
siAGO2-4	NM_012154	5′-GUAUGAGAACCCAAUGUCAUU-3′	Dharmacon	10

Supplementary Table S2. Sequences of Primers Used for RT-qPCR Analysis.

Target	Target NM	Forward	Reverse
SNCA (αSyn)	NM_000345.3	TGCCTGTGGATCCTGACAAT	TACGACTCACTATAGTTCTAGAGGC
SRP54	NM_003136.4	ACACCCGATCCTCTTGCTAC	CAGTACGGATGGTGCCAAAG
AGO2	NM_012154	AGGACTTCAGATGGACTTCCG	ACGGGCATCGAGATCAAGGT
beta-Actin	NM_001101.3	CACCTTCTACAATGAGCTGCG	TAGCACAGCCTGGATAGCAAC
HPRT	NM_000194.3	CTGAGGATTTGGAAAGGGTGTT	ATCTCCTTCATCACATCTCGAG
GAPDH	NM_002046	GTCTCCTCTGACTTCAACAGCG	ACCACCCTGTTGCTGTAGCCAA
OmpA		AACTACTGGAGCTGCCTCG	GCACACACCATCGGCACTC

Supplementary Table S3. Demographic Characteristics of Substantia Nigra Samples

**Diagnosis**	**Age (Years)**	**Sex (M/F)**	**PMI (Hours) ***
PD	78	F	21
PD	79	M	19
PD	88	M	19
PD	77	M	11
PD	82	M	21
PD	75	M	6
PD	79	F	21
Healthy	74	M	16
Healthy	38	M	24
Healthy	83	F	18
Healthy	89	F	5
Healthy	64	M	14
Healthy	87	M	21

* PMI is post-mortem interval
